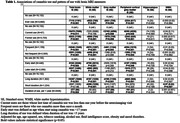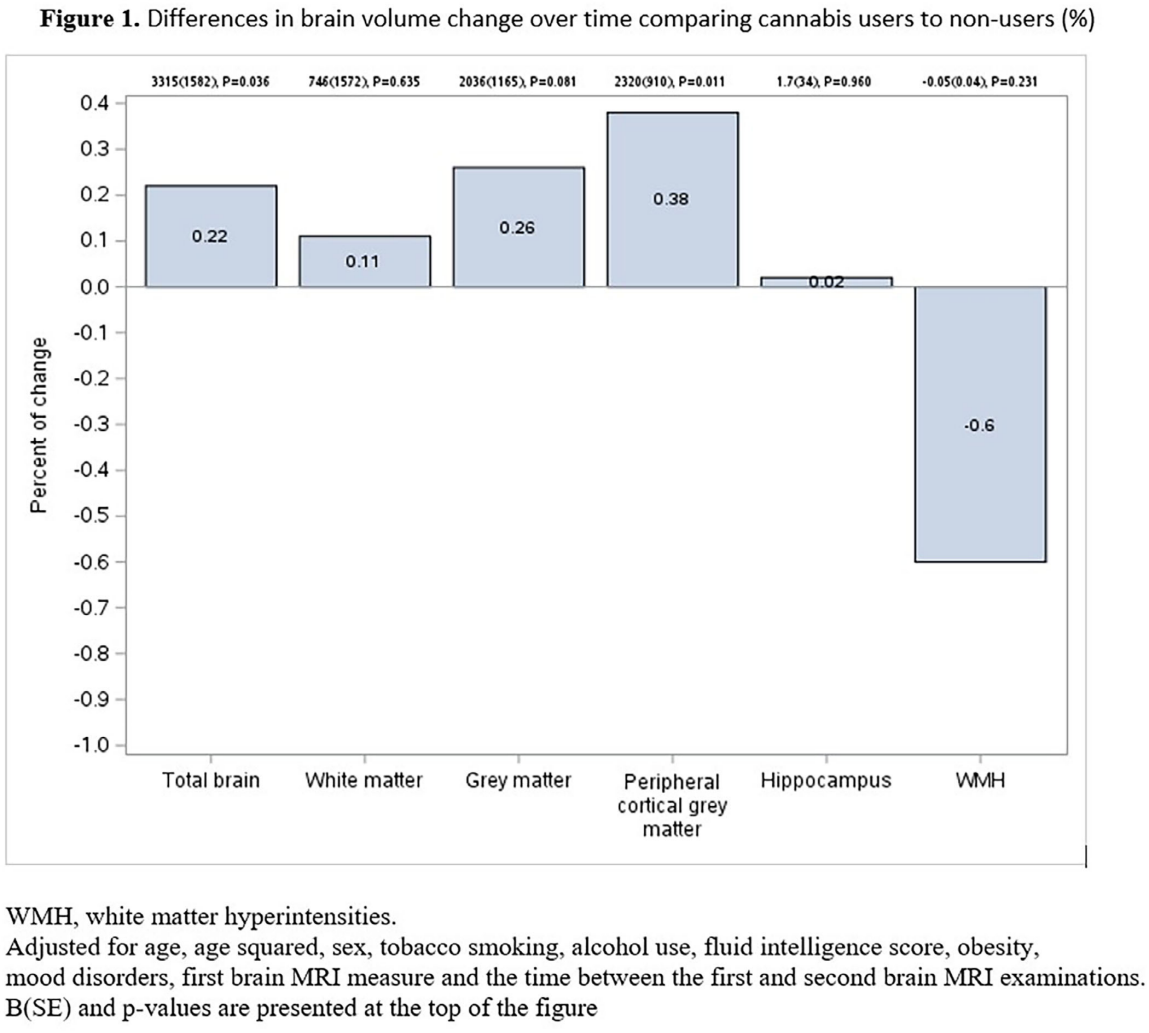# The association between cannabis use and neuroimaging measures in older adults: Findings from the UK Biobank

**DOI:** 10.1002/alz.090279

**Published:** 2025-01-09

**Authors:** Shiraz Vered, Sharon Sznitman, Galit Weinstein

**Affiliations:** ^1^ University of Haifa, Haifa Israel

## Abstract

**Background:**

Cannabis use has increased substantially in recent years, for both recreational and medicinal purposes. While the deleterious effects of cannabis use during adolescence on brain health are well acknowledged, long‐term implications of cannabis use on brains of older adults remain unknown. We explored the associations between past and current cannabis use and volumetric brain MRI measures in older participants of the UK biobank.

**Method:**

This cross‐sectional and longitudinal study (with repeated MRI measures) included dementia‐free UK Biobank participants aged ≥60 years who had data on cannabis use and brain MRI outcomes. Linear regression models were used to assess the relationship of self‐reported cannabis use and patterns of use (i.e., frequency, duration, age of onset) with volumetric brain MRI measures, adjusting for sociodemographic factors and health behaviors. To evaluate change between two MRI examinations, we used the second MRI measures as dependent variables while controlling for the first MRI measures and other covariates.

**Result:**

The sample included 19,932 participants (mean age 68±5 years, 48% men), of which 3,800 (19%) reported lifetime use of cannabis. The cross‐sectional analyses revealed that cannabis users had significantly smaller total brain, white matter, grey matter and cortical grey matter volumes compared to non‐users (b = ‐7110±1181; p<0.001, b = ‐4923±780; p<0.001, b = ‐2031±705; p = 0.004 and b = ‐2334±617; p<0.001, respectively). Stronger associations with brain MRI measures were observed among current vs. former users, and in those who used frequently, started using at younger ages and used for longer time periods (**Table 1**). In contrast, longitudinal analyses showed that cannabis use may be associated with a slower decline in total brain and cortical grey matter volumes (b = 3315±1582; p = 0.036, 0.22% between‐group difference and b = 2320±910; p = 0.011, 0.38% between‐group difference, respectively) (**Figure 1**).

**Conclusion:**

Our study suggests that cannabis use and patterns of use during the life‐course may be related to detrimental brain health effects (e.g. smaller grey and white matter volumes) in middle‐age and older adults. On the other hand, our findings further suggest a possible protective effect (e.g. slower decline in brain volumes) of cannabis use among cannabis users compared to non‐users, which needs to be validated in future research.